# Newborn genetic testing in the United States and access to needed specialist care, National Survey of Children’s Health, 2020: A cross-sectional study

**DOI:** 10.1371/journal.pone.0279352

**Published:** 2022-12-20

**Authors:** R. Constance Wiener

**Affiliations:** Department of Dental Public Health and Professional Practice, West Virginia University, Morgantown, West Virginia, United States of America; Montclair State University, UNITED STATES

## Abstract

Newborn screening tests (NST) are important public health procedures with potential to improve quality of life, and decrease morbidity/mortality by identifying metabolic, genetic, enzymatic, and endocrinological diseases/conditions. In the United States (U.S.), Hawaii conducts the fewest NST (28) and Connecticut conducts the most (75). The purpose of this research is to determine if difficulty receiving specialty care for children with genetic diseases is associated with NST determination of the genetic condition. The research hypothesis is that parents/guardians of children with determination of genetic disease from NST are more likely to report no/slight difficulty accessing specialty care versus parents/guardians of children with genetic diseases whose determination was other than NST. This study has a cross-sectional design with National Survey of Children’s Health, 2020 data. Data were analyzed for frequency, Rao Scott Chi square, and logistic regression analyses. Of 833 children with genetic diseases, most parents/guardians reported no/slight difficulty in receiving needed specialty care; however, children whose determination of a genetic condition was other than NST were 4.82 times as likely (95%CI: 1.66, 14.02; *p* = 0.0040) to have difficulty. In analysis adjusted for sex, race, age, premature birth, and birthweight, the adjusted odds ratio was 6.71 (95% CI:1.91, 23.60 *p* = 0.0031). Parents/guardians of children screened with a positive NST reported less difficulty in receiving needed specialist care as compared with reports of parents/guardians of children with genetic conditions who were diagnosed later. The implication is there would be greater population level benefits realized in the U.S. if NST were expanded in states conducting minimal testing.

## Introduction

Newborn screening tests (NST), which are screening tests for treatable conditions conducted within the first two days of life [1], are successful public health efforts which began in 1963 with phenylketonuria screening [2]. NST have the potential to improve quality of life, and decrease morbidity/mortality by identifying metabolic, genetic, enzymatic, and endocrinological diseases/conditions. The United States (U.S.) Secretary of the Department of Health and Human Services issued the Recommended Uniform Screening Panel (RUSP) list of 35 primary and 26 secondary conditions to be included in NST [3, 4]. The conditions that the Secretary suggests are based, in part, on the ten principles developed by Wilson and Jungner in 1968: the condition is an important health problem whose natural course is understood; there is a recognizable/testable latent/early stage; there is a suitable, acceptable test and treatment with facilities to test and personnel to treat; screening/diagnosis/treatment costs are balanced against medical costs as a whole; and, on-going screenings would occur [5]. These principles have been used to promote expanding NST so that early, specialty care is provided to children in need to reduce morbidity and mortality [6].

There are ethical considerations regarding the benefits and risks of expanding NST to include more genetic screening tests such as protein coding regions of DNA (whole exome sequencing) or the whole DNA strand, (whole genomic sequencing). These genetic screening techniques, once costly, are now similar in cost to other medical tests and may result in early care and ultimately an improved quality of life [7]. Most NST are accurate. For example, in a study of spinal muscular atrophy, researchers found 95% accuracy screening 103,903 newborns [8]. Nevertheless, it is important that parents/guardians are counseled that NST are not diagnostic and there are risks of false positive and false negatives results [4]. With expanding the NST to include the whole genome, there are also risks with secondary use of the sequences and specimens, identification of proband’s family with potential negative consequences such as denial of insurance coverage, denial of employment, privacy issues, and stigmatization. Theoretically, newborn whole genomic screenings benefits outweigh the risk by reducing morbidity and mortality; and laws and practices are in place to limit negative consequences.

In the U.S., NST is the responsibility of states. Most states require testing for the 35 core conditions recommended by the U.S. Secretary of Health and Human Services as well as many of the 26 secondary recommendations. The mean number of NST screening panels in the U.S. is 49.9 (standard deviation, 11.8; minimum, 28 [Hawaii]; maximum, 75 [Connecticut]) [4]. The mean number of NST for states in the Northeast is 55; for states in the Midwest, it is 52; for states in the South, it is 49; and for states in the West, it is 47. These differences are the result of differing strategies, funding, politics, professional groups, policymakers, uncertainty, and diagnostic algorithms [9].

As noted earlier, a key benefit of NST is early, adequate intervention that avoids delays that could result in morbidity or mortality [9]. Also, obstacles to diagnosis and therapies, including access to specialists, may be improved with NST. One consideration for the recommendation to expand NST is a greater likelihood of access to specialty care than would exist if a genetic disease were discovered other than with a NST (clinically). The purpose of this research is to determine if the outcome of difficulty in receiving specialty care for children with genetic diseases is associated with the independent variable of NST determination of the genetic condition. The research hypothesis is that NST determination of a genetic condition is more likely to be associated with reports of no difficulty accessing specialty care for children with genetic conditions as compared with children whose genetic condition determination was other than with a NST.

## Materials and methods

### Ethics

The West Virginia University Institutional Review Board acknowledged this research as non-human subject research (protocol number 2203535297) as it is a secondary data analysis of de-identified publicly available data.

### Population

The data for this research was from the National Survey of Children’s Health, 2020 (NSCH, 2020), available from:

https://www.census.gov/programs-surveys/nsch/data/datasets.html. [10, 11]. The NSCH has a survey frame of all U.S. states and Washington DC with address-based strata for households with children, ages 0–17 years.

### Study design

A multi-stage, stratified sampling approach was used in which approximately 240,000 addresses were randomly selected from the U.S. Census Master Address file and stratified to households likely to have children, and households not likely to have children. State-level samples were created to have approximately the same number of participants from each state. From the strata of households with children, a screening questionnaire either online, on paper or over the phone as presented, and one child in the household was selected as the target for the next survey, the topical questionnaire. Data were collected on 42,777 children in 2020 (weighted overall response rate, 42.4%). There were 90% who received an incentive ($2-$5) to complete the survey. The survey was online, on paper, or via telephone in English/Spanish to address health literacy bias. Methodological details and information about privacy, confidentiality, de-identification, and institutional review board approval are available at: 2020-NSCH-Methodology-Report.pdf (census.gov) [10, 11]. A cross-sectional study design was used following STROBE (Strengthening the Reporting of Observational Studies in Epidemiology) guidelines.

### Study variables

The outcome variable was difficulty in receiving specialist care as perceived by the parent/guardian, based on the question: *“How difficult was it to get the specialist care that this child needed*?*”* The possible responses were “*not difficult*,” “*somewhat difficult*,” “*very difficult*,” and “*it was not possible to obtain care*.” “*Not difficult*,” and “*somewhat difficult*” were recoded as no/slight; and, “*very difficult*” and “*it was not possible to obtain care*” were recoded as yes. The independent variable was NST determination of genetic condition (yes, no), based on the response to: “*Was the [genetic] condition identified through a blood test done shortly after birth*?” [10, 11]. For convenience, if the determination of genetic condition was not by NST, “clinical determination” will be used going forward.

Children were included in the study who: 1) had a genetic condition, determined by endorsing: “*Has a doctor or other health care provider EVER told you that this child has other genetic or inherited condition*; 2) had complete data on the NST question of genetic condition; and 3) had complete data on difficulty in receiving specialist care question.

Other covariates considered as potential determinants: sex (male, female), race (white alone, black alone, other), birth more than three weeks before due date (yes, no), birth weight less than 2,500 grams (yes, no), and age (< 6 years, 6 years to <13 years, 13 years to <18 years). Other social determinants (socioeconomic status, place of residence, education, health insurance, etc.) were not included due to sample size limitations in further parsing the data.

### Statistical analysis

Data were analyzed with SAS version 9.0 (Cary, NC). Provided weights, strata, and sampling unit adjustments were used in the data analyses. Data were analyzed for frequency, and Rao Scott Chi square analyses and logistic regression analyses using the provided sampling weights, and adjusting for the complex study design (strata, cluster and domain). The significance level was set at an alpha of <0.05 for all analyses, including the Likelihood ratio global (omnibus) tests for the logistic regression models; however, as the outcome is binary, a dispersion parameter was not estimated. Ninety-five percent confidence levels were determined for the logistic regression analyses. Epidemiological social determinants (sex, race/ethnicity) and other epidemiological factors impacting healthcare (low birth weight, premature birth) were included in the logistic regression regardless of bivariate Chi square analysis results.

## Results and discussion

The sample had 833 children; mean age, 8.7 years. Most were white children (n = 685; 75.7%, weighted percentage), and female (n = 399; 51.3%, weighted percentage). Approximately one-fifth were born more than 3 weeks before their due date and 14.2% weighed <2,500 grams at birth. There were 128 (11.8%, weighted percentage) who had NST determinations of a genetic condition. Most parents/guardians of children with a genetic condition (n = 759, 88.3%, weighted percentage) reported no/slight difficulty in accessing needed specialty care. Details are provided in Table 1.

**Table 1:** Sample characteristics of children with genetic disease/condition, NSCH, ^a^ 2020 (n = 833).

	Overall		NST determination		Clinical determination	
	Number	Weighted %	Number	Weighted %	Number	Weighted %
	Or Mean (CI)		Or Mean (CI)		Or Mean (CI)	
**Sex**						
Female	399	51.3%	55	12.8, 6.8–18.8	344	87.2, 81.2–93.2
Male	434	48.7%	73	20.7, 13.3–28.2	361	79.3, 71.8–86.7
**Race/ethnicity**						
White alone	685	75.7%	92	12.9, 8.6–17.1	593	87.1, 82.9–91.4
Black alone	44	8.8%	19	57.2, 36.1–78.2	25	42.8, 21.8–63.9
Other	104	15.5%	17	12.1, 2.9–21.2	87	87.9, 78.8–97.1
**Birth more than 3 weeks before due date**						
Yes	123	22.7	33	19.3, 6.7–31.9	90	80.7, 68.0–93.3
No	691	68.0	92	16.4, 10.9–21.9	599	83.6, 78.1–89.1
Missing (19)						
**Birth weight <2500 grams**						
Yes	98	14.2	28	21.9, 6.9–36.8	70	78.1, 63.2–93.1
No	710	85.8	95	15.9, 10.8–21.1	615	84.1, 78.9–89.2
Missing (25)						
**Difficulty receiving specialist care**						
Yes	74	11.7	cell suppressed^c^		66	94.6, 89.7–99.5
No/slight	759	88.3	120	18.2, 12.7–23.6	639	81.8, 76.4–87.3
**Newborn Genetic Screening determination of genetic condition**						
Yes	128	11.8	128	100%	0	
No	705	88.2	0		705	100%
Age in years						
0 to <6	182	30.9	43	18.3, 8.8–27.9	139	81.7, 72.1, 91.2
6 to <13	305	40.5	48	16.5, 8.6–24.4	257	83.5, 75.6–91.4
13 to <18	346	28.6	37	15.2, 7.1–23.3	309	84.8, 76.1–92.9

^a^National Survey of Children’s Health

^b^ Confidence Interval

^c^cells with values <10 are not reported.

Data provided in Table 2 are the relationships of the independent variables with difficulty/no/slight difficulty in receiving needed specialist care. The significant findings were: 1) parents/guardians of children with clinical determination of the genetic condition were more likely to report difficulty in receiving specialist care as compared with parents/guardians of children with NST determination of the genetic condition (*p* = 0.0049); and, 2) parents/guardians of male children were more likely to report difficulty in receiving specialist care (*p* = 0.0058).

**Table 2:** Difficulty receiving needed specialist care among children with genetic disease/conditions vs. variables of interest, NSCH, ^a^ 2020 (n = 833).

	Difficulty in receiving specialist care^b^		No/slight difficulty in receiving specialist care		*p*-value
	Number	weighted %, CI	Number	weighted %, CI	
Sex					**0.0058**
Female	27	5.5, 1.0–10.2	372	94.5, 89.8–99.1	
Male	47	18.2, 9.2–27.1	387	81.8, 72.9–90.8	
Race					0.5980
White alone	57	12.4, 5.8–19.0	628	87.6, 81.0–94.2	
Black alone	cs^c^	15.0, 3.0–27.0	36	85.0, 73.0–97.0	
Other	cs	6.4, 0–13.0	95	93.6, 87.0–100	
Age in years					0.2623
<6	19	8.3, 1.2–15.4	163	91.7, 84.6–98.8	
6 to <13	31	15.9, 4.6–27.2	274	84.1, 72.8–95.4	
13 to <18	24	9.4, 7.0–11.9	322	90.6, 88.1–93.0	
Birth more than 3 weeks before due date					0.1098
Yes	16	21.3, 1.4–41.2	107	78.7, 58.8–98.6	
No	56	9.1, 5.6–12.7	635	90.9, 87.3–94.4	
Birth weight <2500 grams					0.5676
Yes	15	16.2, 1.6–30.8	83	83.8, 69.2–98.4	
No	55	12.0, 5.9–18.0	655	88.0, 82.0–94.1	
Newborn genetic screening determination of genetic condition					**0.0049**
Yes	cs	4.0, 1.0–7.0	cs	96.0, 93.0–99.4	
No	66	13.3, 7.0–19.6	639	86.7, 80.4–93.0	

^a^National Survey of Children’s Health.

^b^The question that was posed was “How difficult was it to get the specialist care that this child needed?” posed to parents/guardians of children who needed care. Responses “not difficult” and “somewhat difficult” were recoded as “no/slight difficulty.” Responses “very difficult” and “it was not possible to obtain care” were recoded as “difficulty.”

^c^cs = cells in the row are suppressed if one of the categories had values <10.

Boldface indicates statistical significance (*p*<0.05).

In the logistic regression analysis, the odds ratio of having difficulty in receiving needed specialist care among children with a clinical determination of the genetic condition was 3.88 (95% confidence interval [CI]: 1.39, 10.84; *p* = 0.0097) as compared to children who had NST determination of the genetic condition in unadjusted analyses. The odds ratio adjusted (AOR) for sex was 4.82 (95%CI: 1.66, 14.02; *p* = 0.0040) and the AOR for adjustment with sex, race/ethnicity, age, premature birth and birth weight was 6.71 (95%CI: 1.91, 23.60; *p* = 0.0031). These results are presented in Table 3.

**Table 3:** Difficulty receiving needed specialist care and newborn genetic screening determination of genetic condition, NSCH, ^a^ 2020.

	Unadjusted odds ratio (95% CI^d^); *p*-value	Model 1^b^ adjusted odds ratio (95% CI); *p*-value	Model 2^c^ adjusted odds ratio (95% CI); *p*-value
Newborn Genetic Screening determination of genetic condition			
Yes	reference	reference	reference
No	3.88 (1.39, 10.84); **0.0097**	4.82 (1.66, 14.02); **0.0040**	6.71 (1.91, 23.60); **0.0031**

^a^National Survey of Children’s Health.

^b^Model adjusted for sex.

^C^ Model adjusted for sex, race/ethnicity, age, premature birth, and birth weight.

^D^ Confidence Interval.

Boldface indicates statistical significance (*p*<0.05).

In this study of children with genetic conditions, the children whose conditions were determined clinically, rather than through NST, were more likely to have difficulty in receiving needed specialist care as compared to children whose genetic conditions were determined via NST. This study highlights one of the benefits of early genetic screening—the easier access to needed specialty. It is the first, to the author’s knowledge, to examine, on a population level, access to needed specialist care among children with genetic conditions. Many of the conditions in NST panels are neurodegenerative requiring timely intervention; therefore, equitable, ethical access to specialist care may make a difference to health and psychosocial outcomes. Several interpretations of the results are plausible. Although NST occurs in every state in the U.S., a genetic condition which could have been determined in a state with core and secondary panels may be missed in a state with limited NST panels, explaining the high number of genetic conditions determined clinically in this study. Alternatively, the genetic conditions may not have a current, acceptable test that could have been administered as an NST. In either case, there would be a delay in diagnosis that parents/guardians may experience as difficulty in accessing needed specialty care. It should be noted, that although clinical determination occurred in many of the cases, the majority of parents in this study did not feel difficulty in receiving specialist care. In the U.S., the American Academy of Pediatrics, the leading professional organization for pediatricians and neonatologists, works to have equitable access and care for all children. This dedication also is a potential factor for the majority of parents in this study to not report difficulty in receiving specialist care for their child/children.

There are few studies with which to compare this study as the literature concerning NST is largely case studies of benefits of genetic NST. For example, a family who consented to have their newborn participate in NST discovered that the newborn was positive for 3-methylcrotomly-coenzyme A carboxylase deficiency which would have resulted in premature death or severe mental/physical deficiencies if not for the early diagnosis and intervention [12].

Of the limited studies of NST outside of case-studies, most involve economics, potential feasibility, and the ethics associated with NST [13, 14]. Critics maintain that low incidence of disorders, treatment barriers, healthcare costs, and inequities raise questions about the worth of investment in NST [15]. In refuting those concerns, researchers showed significant societal benefits from gains in quality-adjusted life-years [16]. Researchers estimated a gain of 85 quality-adjusted life-years savings of $2.4 million per 100,000 infants screened over 60 years in cases of spinal muscular atrophy alone [16]. Others, studying the economic outcome associated with NST for an alpha-glucosidase gene variation, indicated early genetic detection and intervention would have a lifetime increase of 11.66 quality-adjusted life-years and an incremental cost-effective ratio of $379,000/ quality-adjusted life-year as compared with clinical detection [17].

Another group of researchers assessed the current status of NST use. In the U.S., the Secretary of Health and Human Services declined to recommend linking birth certificates with NST, and, as a result, at least 98% of state programs cannot accurately determine NST use/coverage [1]. This is problematic as that data are imperative in assessing the public health impact, quality, and cost effectiveness of the NST [1]. One example cited of the need for such data, pilot research, sharing of research, and taking advantage of progress already made by others was the discovery that cord blood screening was not reliable for metabolic screening and heel stick blood spot screening was a better choice [1]. Large, population-based data are needed for assessing NST. This study takes advantage of a large, nationally recognized data base to evaluate barriers to access to needed care based on NST.

A study limitation is the dependency upon the questions available in NSCH, 2020 for the analyses. Having additional information about the specific genetic conditions, specific needs, age when specialist care was accessed, and availability of specialty services would have enhanced this study. There is a lack of data on the conditions and it is possible that a condition was not serious enough to require a specialist or that the condition was untreatable. The study was limited by the sample size, making statistical analysis by other social determinants of health not feasible, although they were available in NSCH, 2020.

## Conclusion

Parents/guardians of children screened with a positive NST reported less difficulty in receiving needed specialist care as compared with reports of parents/guardians of children with genetic conditions who were diagnosed later. From the second logistic regression model, the results were modified (increased) when sex, race/ethnicity, birth weight, age, and maturity at birth were included. This cross-sectional study of national data from the U.S. indicates greater ease of access to needed care when newborns are identified as potentially having a genetic condition that has a treatment option. Having easy access to a specialist may be vitally important to reduce morbidity and mortality.

## Abbreviations

NST: Newborn screening tests
STROBE: Strengthening the Reporting of Observational Studies in Epidemiology
NSCH: National Survey of Children’s Health
NIH: National Institutes of Health
U.S.: United States; cs: cell suppressed
